# Low temperature-induced DNA hypermethylation attenuates expression of *RhAG*, an *AGAMOUS* homolog, and increases petal number in rose (*Rosa hybrida*)

**DOI:** 10.1186/s12870-015-0623-1

**Published:** 2015-10-05

**Authors:** Nan Ma, Wen Chen, Tiangang Fan, Yaran Tian, Shuai Zhang, Daxing Zeng, Yonghong Li

**Affiliations:** School of Applied Chemistry and Biotechnology, Shenzhen Polytechnic, Shenzhen, Guangdong 518055 China; The Key Laboratory for Quality Improvement of Agricultural Products of Zhejiang Province, School of Agriculture and Food Science, Zhejiang Agriculture & Forestry University, Lin’an, 311300 China; Department of Ornamental Horticulture, China Agricultural University, Beijing, 100193 China

**Keywords:** *Rosa hybrida*, Low temperature, Flower patterning, *RhAG*, DNA methylation

## Abstract

**Background:**

Flower development is central to angiosperm reproduction and is regulated by a broad range of endogenous and exogenous stimuli. It has been well documented that ambient temperature plays a key role in controlling flowering time; however, the mechanisms by which temperature regulates floral organ differentiation remain largely unknown.

**Results:**

In this study, we show that low temperature treatment significantly increases petal number in rose (*Rosa hybrida*) through the promotion of stamen petaloidy. Quantitative RT-PCR analysis revealed that the expression pattern of *RhAG*, a rose homolog of the *Arabidopsis thaliana AGAMOUS* C-function gene, is associated with low temperature regulated flower development. Silencing of *RhAG* mimicked the impact of low temperature treatments on petal development by significantly increasing petal number through an increased production of petaloid stamens. *In situ* hybridization studies further revealed that low temperature restricts its spatial expression area. Analysis of DNA methylation level showed that low temperature treatment enhances the methylation level of the *RhAG* promoter, and a specific promoter region that was hypermethylated at CHH loci under low temperature conditions, was identified by bisulfite sequencing. This suggests that epigenetic DNA methylation contributes to the ambient temperature modulation of *RhAG* expression.

**Discussion:**

Our results provide highlights in the role of *RhAG* gene in petal number determination and add a new layer of complexity in the regulation of floral organ development.

**Conclusions:**

We propose that *RhAG* plays an essential role in rose flower patterning by regulating petal development, and that low temperatures increase petal number, at least in part, by suppressing *RhAG* expression *via* enhancing DNA CHH hypermethylation of the *RhAG* promoter.

**Electronic supplementary material:**

The online version of this article (doi:10.1186/s12870-015-0623-1) contains supplementary material, which is available to authorized users.

## Background

Floral patterning, which is essential for angiosperm reproduction, involves the arrangement of four organs types in concentric whorls: the sepals and petals, which comprise the perianth and form the outer two whorls; and the stamens and carpels, which are the male and female reproductive organs, respectively, and form the inner two whorls. The ABCE model, a broadly accepted model of flower development that was first proposed two decades ago, describes how the combinatorial activity of four classes of homeotic genes determines floral organ identity [1–5]. According to the ABCE model, sepals are specified by A- and E-class genes, petals by A-, B- and E-class genes, stamens by B-, C- and E-class genes, and carpels by C- and E-class genes.

In *Arabidopsis thaliana*, several homeotic genes have been identified, including two A-class genes, *APETALA1* (*AP1*) and *APETALA2* (*AP2*), two B-class genes, *APETALA3* (*AP3*) and *PISTILLATA* (*PI*), one C-class gene, *AGAMOUS* (*AG*), and four E-class genes, *SEPALLATA1* (*SEP1*), *SEP2*, *SEP3*, and *SEP4* [4]. All of the four classes of genes, with the exception of *AP2*, encode MADS-box family transcription factors. These proteins have been proposed to form higher-order complexes that are required for the correct transcription of organ-specific genetic programs [3, 6–9]. Genetic and molecular studies with several model plant species have shown that mutations in A-, B-, C-, and E-class genes all result in abnormal flowers, due to the replacement of one organ type by another [9–11].

A number of ornamental plants, including rose (*Rosa hybrida*), peony (*Paeonia suffruticosa*), carnation (*Dianthus caryophyllus*), and camellia (*Camellia japonica*), have flowers with greater numbers of petals (termed double flowers) and consequently are popular garden plants, due to their attractive appearance. Efforts have been made to characterize the genetic mechanisms involved in the formation of double flowers and studies of various species, including *A. thaliana*, have shown that loss of expression of *AG* results in the conversion of reproductive organs to perianth organs, as well as indeterminacy of the floral meristem, leading to showy double flowers [12–15]. In the ranunculid, *Thalictrum thalictroides*, down-regulation of the *AG* homolog *ThtAG1* has been shown to result in homeotic conversion of stamens and carpels into sepaloid organs, as well as a loss of flower determinacy. Moreover, it was reported that a mutant ThtAG1 protein with K-domain deletions, which was identified in a double-flower ornamental cultivar, cannot interact with the putative E-class protein ThtSEP3, suggesting a deep conservation of the dual function of C-class genes, and of the interactions between C- and E-class proteins in floral patterning [9]. Genetic mapping studies in rose uncovered that the simple versus double corolla phenotype is associated with a single dominant locus, namely *Blfo* or *d6* [16–18], and several QTLs [19–21]. The orthologue of *AGAMOUS* (*RhAG*) was also identified and proved to play an important role in petal doubling in rose, through *RhAG* do not colocalize with *Blfo* or the QTL for petal number [18, 22]. The spatial restriction of the *RhAG* expression domain may result in a homeotic conversion of organ identity from stamens to petals, and is a key factor for selection of double flowers in both the Chinese and peri-Mediterranean centers of domestication [23]. The role of *AG* in the transformation of stamens into petals has been shown to be associated with the A-class gene *AP2* in *A. thaliana*, and the mutual antagonism of *AG* and *AP2* is the central tenet of the ABC model of floral patterning [1]. However, recent research has revealed that the microRNA miR172, which might be *AG*-independent, is a major factor in restricting *AP2* activity, and that whether stamens or petals develop relies on the balance between *AP2* and *AG* activity, rather than a mutual exclusion of the two genes [15].

In addition to genetic determination, petal number in angiosperms is also regulated by phytohormones, including auxin and gibberellic acid [24–26], and by environmental cues, such as light and temperature [24, 27]. For example, early reports demonstrated that either excessively high or low temperatures can cause the malformation of floral organs, especially petals and stamens [24]. Cultivating carnation at a low temperature (5 °C) promoted the formation of secondary growing centers within the flower, and the marked increase in petal number was attributed to the additional petals produced from these centers [24]. In rose, reduced temperature could cause the so-called ‘bullhead’ phenotype, which was accompanied by an increased number of petals and a decreased number of stamens [28–30]. To date, however, little is known about the mechanisms involved in the temperature dependent regulation of petal number. Here, we propose a hypothesis where *AG* genes are involved in the temperature regulated control of petal number and thus in the formation of double flowers.

Roses have been one of the most economically important ornamental plants in the floriculture industry for centuries. As a widespread cut- and cultivated garden flower, the floral pattern is a key trait that determines its ornamental value. In this current study we found that low temperature treatments result in abnormal flowers with more petals than control flowers. We identified a rose C-class gene, *RhAG*, the silencing of which caused an increase in petal number. The overall spatial distribution of *RhAG* transcript in the floral bud was clearly decreased under low temperature conditions and further studies suggested that low temperature exposure caused DNA hypermethylation of the *RhAG* promoter. We conclude that *RhAG* plays an important role in flower patterning and that low temperatures increase the petal number of rose flowers, at least partially, by restricting the expression of *RhAG*.

## Results

### Effects of low temperature on petaloidy of rose stamens

**Fig. 1:**
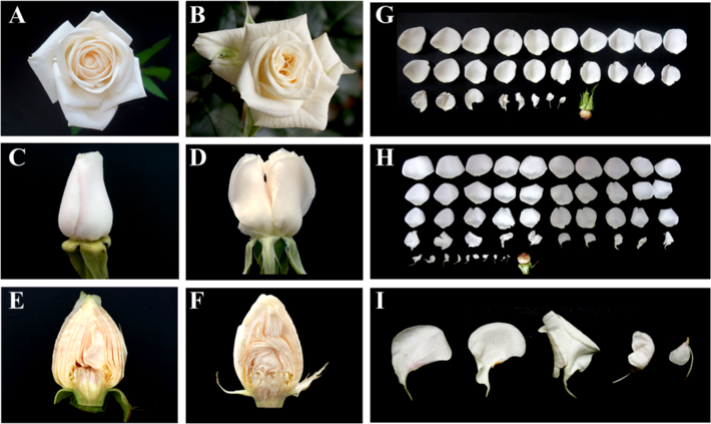
Flower phenotypes of rose plants grown under normal temperature and low temperature conditions. **a**-**b** Top view of rose flowers at stage 9 grown under normal temperature (25/15 °C) (**a**) and low temperature (15/5 °C) (**b**) conditions. **c**-**d** Side view of growing center of rose flowers at stage 9 grown under normal temperature (25/15 °C) (**c**) and low temperature (15/5 °C) (**d**) conditions. **e**-**f** Longitudinal section of rose flowers at stage 6 grown under normal temperature (25/15 °C) (e) and low temperature (15/5 °C) (**f**) conditions. **g**-**h** Petal phenotype of rose flowers grown under normal temperature (25/15 °C) (**g**) and low temperature (15/5 °C) (**h**) conditions. Note that the low temperature treatment resulted in more inner whorl petals. **i** Petaloid stamens

To investigate the effect of low temperature on rose petal development, two-year-old plants were subjected to different temperature treatments: 25/15 °C (day/night, hereafter), 20/10 °C and 15/5 °C. We found that, compared with the control treatment (25/15 °C), a lower temperature regime of 20/10 °C or 15/5 °C resulted in the formation of deformed flowers, which had irregular shape in flower center (Fig. 1d). As shown in Fig. 1a-d, the typical phenotype of these flowers was two growing centers and a larger flower size than the control (Additional file 1: Figure S1). Longitudinal sections of the flowers showed that the petal shape of the deformed flowers was clearly irregular, unlike that of the normal flowers (Fig. 1e, f).

The proportion of deformed flowers was only approximately 2 % at the control temperature, but was 36 % at 20/10 °C and 88 % at 15/5 °C, indicating that the occurrence of deformed flower formation increased markedly at lower temperatures (Additional file 1: Figure S2). Generally, the modern cultivated rose has double flowers and we investigated whether the larger flower size resulting from the treatments was a result of the petal size and/or number. Rose flowers at stage 7 (completely opened bud; Please see Methods for details) exposed to each treatment were dissected and floral organs (sepals, petals, stamens and carpels) were counted. Neither sepal nor carpel number was significantly different between the three treatments (Table 1), but the number of petals showed a marked increase under low temperature conditions. At the control temperature, the average number of petals was 28, indicating that the flowers are semi-double flowers (8 to 40 petals) [23]. However, at a lower temperature of 20/10 °C, the petal number significantly increased to 33, and at the lowest temperature to 49, turning the flowers from semi-double flowers into double flowers (i.e. >40 petals). Interestingly, the decrease in temperature also reduced the number of stamens as the average stamen number was 126 at the control temperature and decreased to 121 and 106 following 20/10 °C and 15/5 °C treatments, respectively. Generally, rose flowers have a few internal petals that have a slender base and an irregular shape, suggesting a homeotic conversion from a stamen into a petal (Fig. 1g-i), and it is therefore noteworthy that the number of petal/stamen chimeras in low temperature treated flowers was higher than in the control flowers, thereby contributing to an increase in total petal number (Fig. 1g, h). These results indicated that there might be a gradual transition of petaloid stamens from outer whorls to inner whorls even in normal cultivated rose flowers, but low temperature could enhance the formation of petaloid stamens (Fig. 1g, h).

**Table 1:** Numbers of floral organs from flowers grown at different ambient temperatures

Treatment	Sepals	Petals	Stamens	Carpels	Total
25/15 °C	5.0 ± 0.0 a	28.3 ± 2.6 c	126.0 ± 17.2 a	116.3 ± 15.7 a	275.6 ± 31.7 a
20/10 °C	5.0 ± 0.0 a	33.1 ± 3.0 b	120.9 ± 9.4 a	113.2 ± 7.4 a	272.1 ± 10.8 a
15/5 °C	5.0 ± 0.0 a	48.5 ± 5.4 a	105.8 ± 18.6 b	118.6 ± 14.5 a	278.0 ± 25.6 a

Values are means ± SD. Lower-case letters indicate significant differences according to the Duncan’s multiple range test (*p* < 0.05)

It is also worth noting that total number of floral organs is not significantly different among treatments (Table 1). Therefore, low temperature has not indeterminacy effect on flower development. Taken together, these results suggest that low temperature exerts a strong influence on petaloidy of rose stamens.

### Expression analysis of *RhAG* gene in response to low temperatures during flower development

**Fig. 2:**
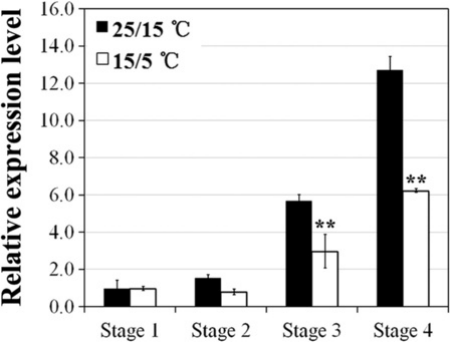
Expression pattern of *RhAG* in response to low temperatures during flower development. Expression of *RhAG* was monitored by quantitative RT-PCR. Two-year-old rose plants were cultivated at 25/15 °C (black column) or 15/5 °C (white column). Flowers were collected at stage 1 to stage 4. *RhTCTP* was used as an internal control for all the tested genes. The expression level of *RhAG* at stage 1 grown at 25/15 °C was defined as 1.0. Values are means ± SD (*n* = 3). Asterisks indicate significant differences calculated using the *t* test (**p < 0.01; *p < 0.05)

It has been reported that *AG* gene plays a dual role in specifying reproductive organ identity and floral meristem determinacy [9], as well as being involved in the determination of petal number [23]. To understand whether *AG* gene is associated with the low-temperature-regulated petal doubling in rose, we analyzed the effect of low temperature on the expression of *RhAG*, a rose homolog of *AG* [23], during rose flower development through quantitative RT-PCR. Results showed that the expression level of *RhAG* was low at stages 1 and 2, when the sepal and petal primordia form, before rising markedly at stages 3 and 4, when the stamen and carpel primordia form (Fig. 2). This expression pattern correlates with its classification as a C-class gene. Furthermore, low temperature exposure significantly decreased its expression level at the stamen and carpel formation stages, suggesting that *RhAG* may be involved in the low temperature induced homeotic conversion of stamens into petals.

### Flower patterning of *RhAG*-silencing flowers phenocopies low temperature treated flowers

**Fig. 3:**
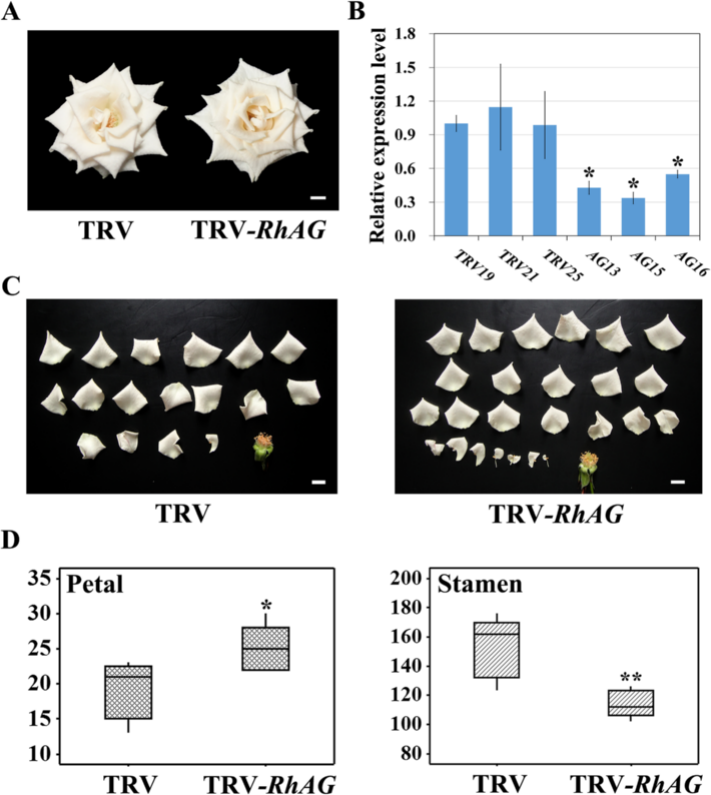
Silencing of *RhAG* in rose flowers. Three month old rose plants were infiltrated with *A.tumefaciens* containing a TRV control (TRV, pTRV1 + pTRV2), or TRV carrying an *RhAG* fragment (TRV-*RhAG*, pTRV1 + pTRV2- *RhAG*). **a** Phenotype of the TRV control (*left*) and *RhAG*-silenced (*right*) flowers at stage 10. **b** Quantitative RT-PCR analysis of *RhAG* expression in TRV control and *RhAG*-silenced petals. The expression level of *RhAG* in the TRV19 control was set to 1.0. *RhTCTP* was used as the internal control. Floral buds at stage 3 were used. Values are means ± SD (*n* = 3). **c** Petals from TRV flowers (*left*) and *RhAG*-silenced flowers (*right*). **d** Petal (*left*) and stamen (*right*) numbers of TRV flowers and *RhAG*-silenced flowers (*n* = 5). *Asterisks* indicate significant differences calculated using the *t* test (**p < 0.01; *p < 0.05). Scale bars = 1 cm

Since the expression of *RhAG* was reduced under low temperature conditions, we looked for evidence of causal relationship between *RhAG* function and the low temperature induced increase in petal number, by silencing *RhAG* in three month old rose plants using virus-induced gene silencing (VIGS). Floral phenotypes indicative of gene silencing were observed in fully opened infiltrated plants (Fig. 3a, b). At stage 10 the yellow anthers of the TRV-infected flowers were visible. In contrast, the anthers of the *RhAG*-silenced flower were not exposed as they were covered with the additional internal petals. We counted the number of petals and stamens of flowers from both groups and found that the petal number of the silenced flowers was substantially higher, while the stamen number decreased compared with the TRV controls (Fig. 3c, d). TRV flowers had an average of 19 petals, while *RhAG-*silenced flowers had 25. Conversely, the average stamen number in *RhAG-*silenced flowers was 25 % lower than that of the TRV controls. Furthermore, compared to TRV-treated flowers, the *RhAG*–silenced flowers had more petaloid stamens (Fig. 3c), as was observed in the low temperature treated flowers. Interestingly, we found that partial gynoecia in *RhAG*-silencing flower were converted to sepal-like organs, indicating that *RhAG* was also involved in identity of gynoecia (Additional file 1: Figure S3). These results suggested that *RhAG* plays an important role in rose flower petal number control and homeotic conversion of stamens into petals.

### Expression pattern of *RhAG* in floral primordia during low-temperature-responsive rose flower development

**Fig. 4:**
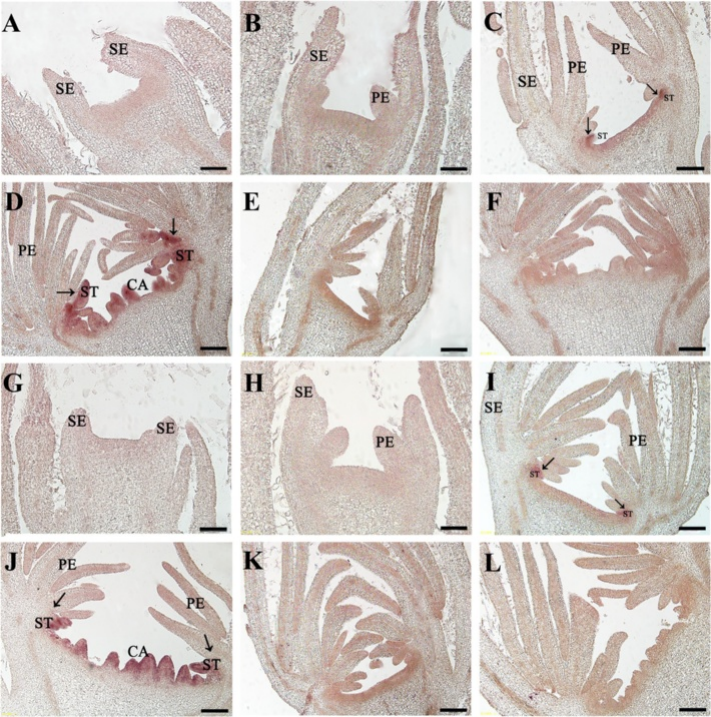
*In situ* hybridization of *RhAG* mRNA accumulation in rose flower buds. *RhAG* was detected using a DIG-labeled probe in flower buds of rose plants grown under normal temperature (25/15 °C) (**a-f**) or low temperature (15/5 °C) (**g-l**) conditions. **a** and **g**, stage 1 floral buds; **b** and **h**, stage 2 floral buds; **c** and **i**, stage 3 floral buds; **d** and **j**, stage 4 floral buds. A sense probe was used for stage 3 (**e** and **k**) and stage 4 (**f** and **l**) buds as a negative control. SE, sepal; PE, petal; ST, stamen; CA, carpel. Arrows show the boundary between petal and stamen. Scale bars = 100 μm (**a**, **b**, **g** and **h**) or 200 μm (**c**-**f**; **i**-**l**)

To further confirm the proposed role of *RhAG* in the regulation of flower development, we examined its expression pattern in the floral primordia of low temperature treated flowers by *in situ* hybridization. As expected, irrespective of the ambient temperature regime or development stage, the expression of *RhAG* was undetectable in whorls 1 and 2 (Fig. 4a-d and g-j), while a persistent signal was detected in whorls 3 and 4 once they emerged (Fig. 4c, d, i, and j), consistent with previous reports [23]. Interestingly, we observed that the expression pattern was different at stage 4 between low-temperature treated buds and controls. In low-temperature treated buds, which developed more petal primordia, the expression area of *RhAG* was restricted towards the center of the meristem, which might give rise to the fourth whorl, and extended slightly to the lateral area where only a few stamen primordia emerged (Fig. 4j). In control flowers, the *RhAG* signal extended to a wider domain of whorl 3, from which many stamen primordia emerged (Fig. 4d). The reduced expression area supported the data obtained by quantitative RT-PCR and further suggested that the restricted expression of *RhAG* caused by the low temperature treatments might promote the petaloidy of stamens, resulting in the formation of double flowers.

### Low temperate induces DNA hypermethylation in the *RhAG* promoter

Over the last decade, there has been growing evidence that DNA methylation is involved in plant responses to various environmental stimuli [31]. Previously, *AG* gene of *A. thaliana* was reported to be hypermethylated in an antisense-*MET1* transgenic line [32].

**Fig. 5:**
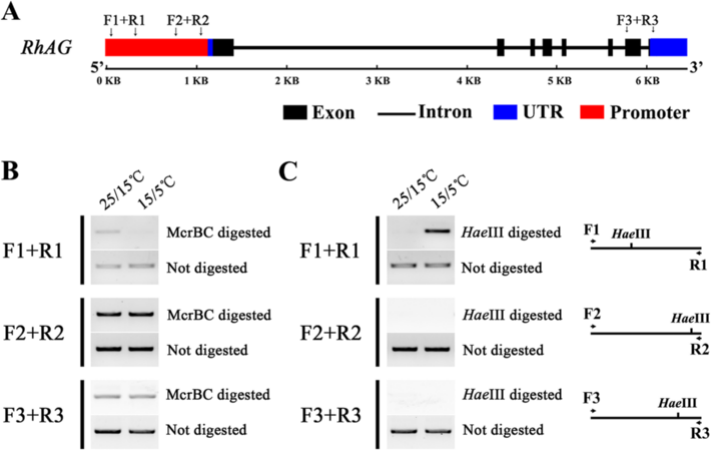
Effects of low temperature on DNA methylation of the *RhAG* gene. The effects of low temperature (15/5 °C) on cytosine DNA methylation in the *RhAG* gene (**a**) were determined by *McrBC* digestion (**b**) and *Hae*III-mediated Chop-PCR assays (**c**). **a** Schematic structure of the *RhAG* gene. The primers used for the Chop-PCR assay are indicated as F1 + R1, F2 + R2, and F3 + R3. **b** *McrBC* digestion assay. Genomic DNA was digested with *McrBC* for 3 h and amplified by PCR. **c** *Hae*III-mediated Chop-PCR assay. Linearized genomic DNA was digested with *Hae*III for 3 h and amplified by PCR. In each assay, undigested genomic DNA was used as a control

We hypothesized that the observed reduction in *RhAG* expression at low temperatures might be related to a change in DNA methylation status. To test this we firstly cloned the genomic sequence and a 1,182 bp-DNA sequence upstream from the start codon of *RhAG* from rose flowers. The gene structure consists of eight exons and seven introns, with a total length of 4,942 bp (Additional file 1: Figure S4). Then the 1,182 bp-fragment upstream from *RhAG* was digested by the McrBC restriction enzyme that cleaves methylated DNA, no matter it is CG, CHG or CHH methylation [33]. Three regions were then chosen for PCR amplification: −1,149 to −826 bp (F1 + R1); −446 to −48 bp (F2 + R2); and +4493 to +5058 bp (F3 + R3) (Fig. 5a). Figure 5b shows that the F1 + R1 region of the *RhAG* promoter was heavily methylated when the rose plants were exposed to the low temperature condition 15/5 °C, while the two other regions did not exhibit obvious differences in DNA methylation between growth at a normal temperature (25/15 °C) and the low temperature (15/5 °C). Since several digestion sites of *Hae*III, a CHH (H represents A, T, or C) locus methylation-sensitive enzyme, were identified in the *RhAG* promoter, we further tested its methylation level by digestion with *Hae*III, followed by PCR (Chop-PCR) [33]. We found that the low temperature condition led to CHH DNA hypermethylation in the tested F1 + R1 region, which was thus resistant to *Hae*III cleavage (Fig. 5c).

**Fig. 6:**
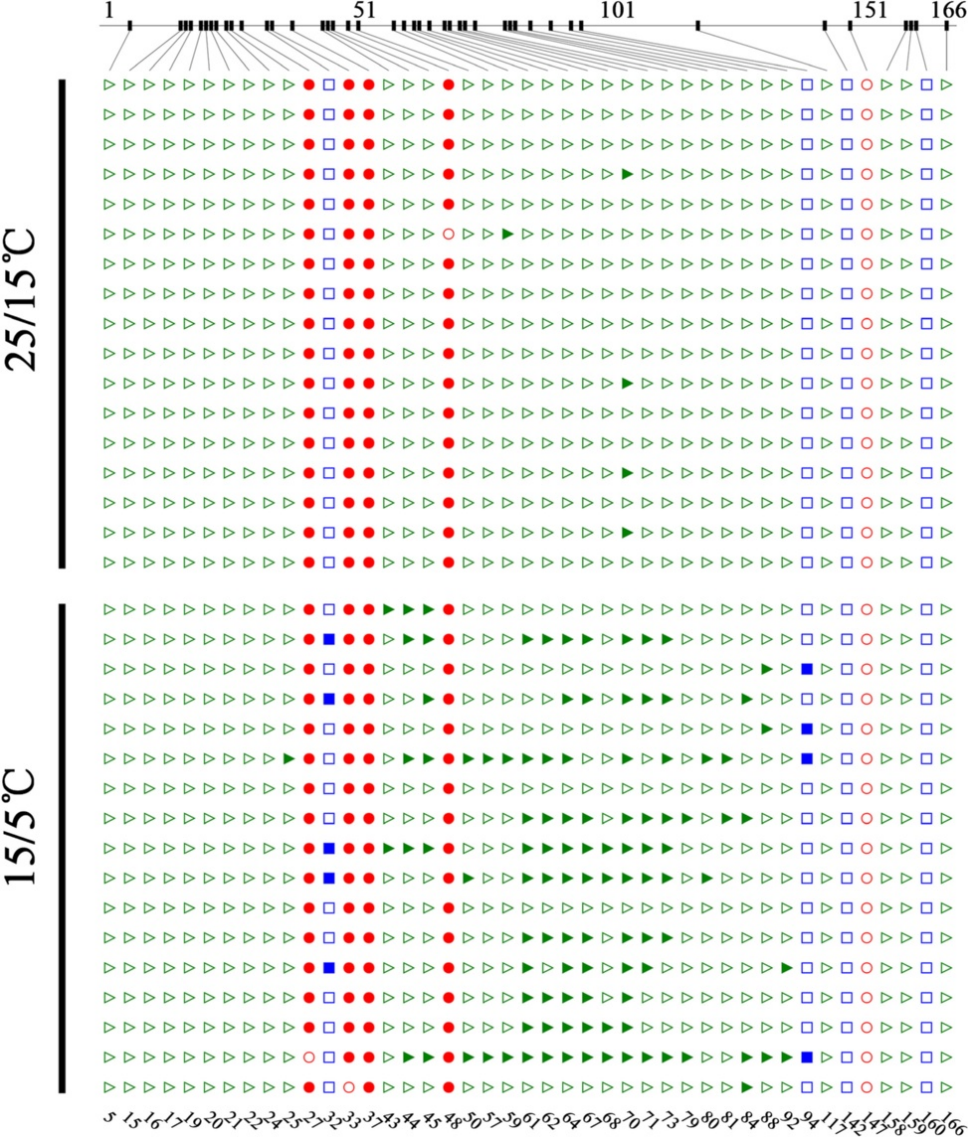
DNA methylation assay of the *RhAG* promoter by bisulfite sequencing. Genomic DNA was isolated from flower buds grown under normal temperature (25/15 °C) or low temperature (15/5 °C) conditions. Bisulfite-converted DNA was amplified and sequenced. The sequences were analyzed using the CyMATE programme [58]. The 166 bp DNA sequence analyzed by bisulfite sequencing is presented as top diagram. The number on the top indicate the base pair in the sequence; the number on the bottom indicate the cytosines in the sequence. Green triangles, blue squares and red circles represent cytosines in CHH, CHG and CG (H represents A, T, or C) configurations, respectively. Filled shapes indicate methylated sites, while open shapes indicate sites that are not methylated. Seventeen clones were analyzed for each treatment. The cytosines density is indicated by connection lines at the top panel as described in Hetzl et al. [58]

We next examined the methylation status of the −1,149 to −826 bp region by bisulfite sequencing using sequencing primers designed with MethPrimer (http://www.urogene.org/cgi-bin/methprimer/methprimer.cgi) [34]. According to sequencing results, we found that methylation status was changed under low temperature in a 180 bp-in-length fragment ( −1,042 to −863 bp). In this region, almost all CG loci were methylated and the methylation level was nearly the same in plants grown under normal or low temperature conditions (Fig. 6; Additional file 1: Figure S5A). All four CHG (H represents A, T, or C) loci were non-methylated under normal temperature conditions, while two CHG loci were methylated when plants were grown at low temperatures, although the methylation percentage was only ~20 % (Fig. 6; Additional file 1: Figure S5B). As expected, the largest difference in DNA methylation was found in the CHH loci, which were almost all non-methylated at the normal temperature, while 20 out of 34 were hypermethylated at the low temperature. In addition, all CHH loci that showed a change in DNA methylation status were clustered (Fig. 6; Additional file 1: Figure S5C). Given that DNA hypermethylation of promoters usually correlates with repression of gene expression [31], these results support the hypothesis that the low temperature induced reduction in the expression of *RhAG* is, at least in part, a result of CHH DNA hypermethylation of the *RhAG* promoter.

## Discussion

### Effects of low temperature on petal doubling through stamen petaloidy

Flowering is an essential phase of angiosperm reproduction and continuation of the species. The flowering process is controlled by various environmental factors, such as ambient temperature, which is known to influence the rate of flower development and flower quality. For example, low temperatures during the early stages of flower development can delay flower bud initiation and development in some cultivars of rose (*Rosa hybrida*), lily (*Lilium hansonii*), and chrysanthemum (*Dendranthema morifolium*) [35–37]. Temperature also regulates floral organ identity, resulting in homeotic transformation between different whorls and changes in organ numbers. In rose, high temperatures can cause the transformation of reproductive organs into leaf-like organs [27], and petal number has also been reported to be regulated by either high or low temperatures in several species. One example is from carnation, where Holley and Baker [38] reported that the petal number of some cultivars was reduced at high temperatures. This contrasts with the findings of Garrod and Harris [24], who suggested that a low temperature (5 °C) promotes the formation of secondary growing centers, producing additional petals and hence a marked increase in total petal number. This latter result is consistent with studies of rose flowers, where it was found that the “bullhead” phenotype with increased number of petals and decreased number of stamens was induced by low temperatures and substantially decreased when the temperature was increased [28–30, 39].

In this study, we demonstrated that flowers of rose *R. hybrid* cv. Vendela exposed to low temperatures have a greater total number of petals and a larger flower bud than flowers grown at normal growth temperatures, in agreement with a previous study [39]. Moreover, we found that the increase in the number of petals was accompanied by a decrease in stamen number, while the total number of both petals and stamens was similar between the flowers from plants grown at different temperatures. We also observed that more petaloid stamens were formed in the low temperature treated flowers. One explanation for these phenotypes is that low temperatures cause increase of petal numbers, at least in part through stamen petaloidy. In addition, we observed that some plants had sepal-like organs in the center of the flower at low temperatures, suggesting a homeotic transformation of carpels to sepals, which is consistent with previous reports in *A. thaliana* [10].

Interestingly, a previous report showed that in *Rosa hybrida* cv. Motrea, high temperature regime (26/21 °C, day/night) elevated the proportion of flowers exhibiting phyllody phenotype to four times higher than low temperature (21/15 °C). The petal number of phyllody-phenotype flowers was higher than normal flowers, under either 26/21 °C or 21/15 °C condition, and the increase of petal number was positively correlated with the extent of phyllody phenotype, more severe phenotype with more petals [27, 40]. Reduced cytokinin content was considered to be responsible to the phyllody phenotype [27]. These reports implied that petal number might be controlled by several pathways and more studies should be conducted to clarify it in the future.

### Involvement of *RhAG* in petal number increasing at low temperatures

The floral homeotic C-class gene *AG* has a dual role in regulating floral meristem determinacy and reproductive organ identity, which is strongly conserved even in distantly related angiosperms, including the model plants *A. thaliana*, *Antirrhinum majus*, and the ranunculid *T. thalictroides* [9, 41, 42]. These studies suggest that plants with reduced *AG* function convert reproductive organs into perianth organs and develop indeterminacy of the floral meristem, causing double-flowers with excess petals. In rose, expression of *RhAG* was reported to be associated with selection of cultivars with higher petal numbers during domestication in Europe/Middle East and in China [23]. Among roses with similar genetic backgrounds, the expression domain of *RhAG* is restricted toward the center of the flower and is clearly narrower in double-flowered roses than in simple flowered cultivars. Moreover, this border of the *RhAG* expression domain is labile, allowing the selection of rose flowers with increased petal number [23].

Based on the above studies, we hypothesized that the rose flower with extra- petals-phenotype induced by low temperatures is related to *AG* expression, and so we examined the expression of *RhAG* and other homeotic genes in rose plants grown at low temperatures. Expression analysis showed that, as expected, the expression of *RhAG* was significantly down-regulated by low temperature treatments at the stamen and carpel formation stages. To obtain additional genetic evidence, we suppressed the expression of *RhAG* in rose plants using VIGS. This resulted in additional petal formation in the flower buds, a reduced number of stamens and an increased number of petaloid stamens. We noticed that the average petal number in the TRV control flowers in VIGS experiment (Please see Fig. 3d) was substantially lower than in the control flowers in low temperature treatment experiment (Please see Table 1). This phenomenon is likely due to the variability of flowers during different seasons, batches, ages of seedlings as well as viral effects, since it has previously been reported in ranunculid species that TRV2-empty plants showed asymmetric reduction in sepal size, occasional brown, necrotic spots on sepal, as well as stunted growth. However, the stunted growth did not affect subsequent growth [9, 43]. Whether such virus-induced effects existed in the TRV2-empty rose plants in this current study is unclear. In addition, the total number of petal plus stamen was less in *RhAG*-silenced flowers than in TRV controls, while it was not influenced in the low temperature test. This could be an environmental effect due to different growth conditions, such as seasons. Regardless, the difference in petal and stamen numbers between *RhAG*-silenced flowers and TRV controls indicates that low temperature can regulate petal numbers at least in part by a homeotic conversion of stamens into petals, *via* suppressing the expression of *RhAG*. Finally, we also found sepaloid organs in the center of *RhAG*-silenced flower, where carpels normally form. This phenotype was not observed in the TRV control flowers and was similar to the phenotype of low-temperature treated flowers, further supporting the proposed role of *AG* in determining reproductive organ identity and the reliability of the VIGS experiments.

We also conducted an *in situ* hybridization study of rose buds to determine the expression pattern of *RhAG* in response to growth at low temperatures, and observed that under such conditions *RhAG* was expressed in a reduced area toward the center of the flower. The change of the spatial pattern of expression of *RhAG* in response to low temperatures might promote the formation of double-flowers, as has been proposed as a mechanism underlying the selection of double-flowers during rose domestication [23].

Since determination of floral organ identity requires the involvement of multiple floral homeotic genes and other regulatory factors during floral development, it is possible that low temperature also influence the expression of other homeotic genes, which might contribute to double-flower formation. The identity of other genes involved in this process, especially those related to petal and stamen primordia formation, is an interesting subject for future research.

### Involvement of epigenetic DNA methylation in *RhAG* regulation

It has been well documented that epigenetic DNA methylation is involved in many aspect of plant development, as well as in responses to endogenous and exogenous cues [31, 44–46]. In *A. thaliana*, DNA methylation could occur in the contexts of CG, CHG, and CHH (H = A, C, or T) in plants. Once established, CG and CHG methylation is maintained by MET1 and CMT3, respectively, whereas CHH methylation needs to be established *de novo* by DRM2 and CMT2 during every cell cycle [47, 48]. For gene expression, the most important factor is the region where DNA methylation occurs instead of DNA context. If DNA methylation occurs in the promoter region of a certain gene, no matter it is CG, CHG and CHH methylation or both, it will cause gene silencing. However, DNA hypermethylation in gene body usually represent a feature of transcribed genes [47, 49–51]. Here, we report that DNA methylation of the promoter of rose *RhAG*, a homolog of *A. thaliana AG*, is regulated by ambient temperatures. *McrBC* and *Hae*III-digestion indicated that growth at low temperatures (15/5 °C) resulted in heavier methylation in the *RhAG* promoter when compared to the plants grown at normal temperatures (25/15 °C). Bisulfite sequencing further indicated that CHH DNA methylation was highly induced by low temperatures. Interestingly, only ~50 % of the CHH loci in the tested region were methylated, suggesting that low temperature induced CHH methylation is site-specific. Strikingly, *AG* gene could be hypermethylated in antisense-*MET1* transgenic *A. thaliana* lines. In addition, most methylated sites in tested regions were CHH, while *MET1* is considered to be responsible for CG loci methylation [32]. Thus, epigenetic regulation of *AG* gene might be a conserved pathway. Generally, DNA methylation near gene promoters is considered to correlate with repression of gene expression [47, 49–51]. In tobacco, cold stress activated gene expression of *NtGPDL via* an induction in DNA demethylation in the coding region [52]. Thus, our results suggest that ambient temperature triggers complex changes in the DNA methylation status of *RhAG* to regulate its expression level in a conserved manner. The nature and dynamics of these modifications, as well as the identification of possible regulators, will be the subject of future studies.

## Conclusions

In present work, we found that *RhAG*, an *AG* homolog in rose, regulated petal number in an ambient temperature-dependent manner. This is based on several lines of evidence. First, low temperature treatment significantly increases petal number in rose through the promotion of stamen petaloidy. Second, quantitative RT-PCR analysis revealed that the expression pattern of *RhAG* is associated with low temperature regulated flower development, and the silencing of *RhAG* caused an increase in petal number through an increased production of petaloid stamens. Third, *in situ* hybridization studies showed the overall spatial distribution of *RhAG* transcript in the floral bud was clearly decreased under low temperature conditions. Fourth, analysis of DNA methylation level showed that low temperature treatment enhances the methylation level of the *RhAG* promoter, and a specific promoter region that was hypermethylated at CHH loci under low temperature conditions was identified by bisulfite sequencing. In summary, our results provided new insights into the underlying mechanism of ambient temperature-regulated flower patterning. And we demonstrated that low temperature probably attenuated *RhAG* expression at least partially via enhancing DNA CHH hypermethylation of the *RhAG* promoter.

## Methods

### Plant materials

Rose (*Rosa hybrida*) cv. Vendela plants were grown in a greenhouse at the Shenzhen Polytechnic. Flower development in roses includes early development stages [23] and opening stages [53]. We divided the whole process of flower development into 11 stages: stage 1, sepal primordia emerge and develop; stage 2, petal primordia emerge and develop; stage 3, stamen primordia emerge and develop; stage 4, carpel primordia emerge and elongate; stage 5, floral bud differentiation is complete but the bud is not open; stage 6, bud is partially open; stage 7, bud is completely open; stages 8 and 9, flower is partially open; stage 10, flower is fully open with anther appearance (yellow); and stage 11, flower is fully open with anther appearance (black).

### Low temperature treatment of flowers

Two-year old rose plants were pruned uniformly before treatment. All plants were then cultivated in three controlled environment chambers (Thermoline TPG-6000-TH) with day/night temperature regimes of 25/15 °C (control), 20/10 °C and 15/5 °C. For each treatment, around 30 plants were used. All the three treatments were under a 12/12 h light/dark cycle, 70–80 % relative humidity and 850 μmol m ^−2^ s ^−1^ light intensity. For the plants subjected to 20/10 °C and 15/5 °C, once the flower bud differentiation was complete the chamber temperature was changed to the normal growth temperature of 25/15 °C.

### Observation of flower phenotypes and floral organ counts

Flower phenotypes were observed after flower development stage 6 and floral organs were counted at stage 7. Fifty flowers from each treatment were selected randomly (only one or two flowers were used from each plant), and the proportion of deformed rose flowers was recorded. Fifteen flowers from each treatment were dissected and 4 whorls of organs (sepals, petals, stamens and carpels) were counted. And petal/stamen chimera was counted as petal. The statistical significance was analyzed using a one-way ANOVA or Student’s *t* test.

### RNA extraction

For quantitative RT-PCR analysis, floral buds of plants subjected to a cold treatment (15/5 °C) or grown in control conditions (25/15 °C) were sampled at stages 1, 2, 3 and 4. Whole buds were harvested and frozen in liquid nitrogen before storage at −80 °C. Total RNA was extracted using the Trizol reagent (Life Technologics, Ohio, USA) according to the manufacturer’s instructions.

### Quantitative RT-PCR

For quantitative reverse transcriptase polymerase chain reaction (quantitative RT-PCR), complementary DNAs (cDNAs) were synthesized from 2 μg total RNA using the SuperScript™ II RNase H reverse transcriptase (Invitrogen). One μl cDNA samples were used as the template in a 20 μl PCR reaction using the ABI PRISM® 7500 Sequence Detection System in standard mode with the Platinum SYBR Green qPCR Supermix-UDG (Invitrogen). The primers used are listed in Additional file 1: Table S1. *RhTCTP* was used as an internal control. The expression levels were analyzed using the relative standard curve method normalized to *RhTCTP*. All reactions were performed for three biological replicates.

### *RhAG* promoter isolation

For isolation of 5’ upstream sequences, including promoter and 5’ UTR of *RhAG*, hi-TAIL PCR was performed as in Liu and Chen [54].

### *RhAG* silencing in rose flowers

Since the efficiency of original TRV vector was relative low in rose plants, we conducted the VIGS experiments by using a modified and GFP-labeled TRV-GFP system [55]. The silencing of *RhAG* in rose flowers by VIGS was performed as previously described with some modifications [55, 56]. For silencing of *RhAG*, a 332-bp fragment at the 3’ UTR was chosen and inserted into the pTRV2 vector. For vacuum infiltration, three-month-old rose plants were immersed in the bacterial suspension solution and infiltrated under vacuum at 0.9 atm. Then plants were washed and cultivated in MS liquid medium at 23/18 °C. After the plants had rooted they were transplanted into pots and grown in controlled environment chambers at 25/15 °C, 70-80 % relative humidity and 850 μmol m ^−2^ s ^−1^ light intensity, under a 12/12 h light/dark cycle. When floral buds emerged, stage 3 buds were pre-screened by detecting GFP fluorescence, and the positive ones were sampled for RNA extraction. The expression level of *RhAG* was determined by quantitative RT-PCR and floral phenotypes of the selected plants were also observed.

### *In Situ* hybridization

Tissue fixation and *in situ* hybridization were performed as in Zhang et al. [57], Ma et al. [56] and Dubois et al. [23]. To synthesize the *in situ* probes, PCR amplification of cDNA was conducted using gene-specific primers containing the T7 and SP6 RNA polymerase binding sites [56]. T7 RNA polymerase was used to generate the antisense probes and SP6 RNA polymerase for sense probes. The primer pairs are listed in Additional file 1: Table S1.

### Chop-PCR and bisulfite sequencing PCR

DNA methylation status was analyzed by Chop-PCR and bisulfite sequencing [33]. For Chop-PCR, genomic DNA (500 ng) was digested with the methylation-sensitive restriction enzyme *Hae*III or the methylated DNA-digesting enzyme *McrBC* for 3 h. The digested DNA was used as a template to amplify the *RhAG* promoter and undigested genomic DNA was amplified as a control. For bisulfite sequencing, the primers were designed by MethPrimer (http://www.urogene.org/cgi-bin/methprimer/methprimer.cgi) [34]. Genomic DNA was isolated from a pool of ten flower buds grown under normal temperature (25/15 °C) and low temperature (15/5 °C) conditions, before being subjected to bisulfite treatment using an EZ DNA Methylation-Gold kit (Zymo Research, Orange, CA, USA) according to the manufacturer's instructions. For each treatment, 17 clones were chosen randomly and sequenced. The sequences were aligned using the ClustalW server (http://www.ch.embnet.org/software/ClustalW.html) and then analyzed using the CyMATE program (http://cymate.org/cymate.html) [58].

## Availability of supporting data

The complete coding sequence and the promoter of *RhAG* is available at NCBI under accession number U43372 and BankIt1849645 Seq1 KT429820, respectively.

## Additional file

Additional file 1: Figure S1. Effect of low temperature on flower diameter (mm) at different flower developmental stages. Data represents means from 5 replicates ± SD. RT, regular temperature (25/15 °C; day/night); LT, low temperature (15/5 °C; day/night). **Figure S2.** The proportion of deformed rose flowers from plants grown under different temperature conditions. Two year old plants were pruned uniformly and exposed to day/night temperature regimes of 25/15 °C (control, left), 20/10 °C (middle) or 15/5 °C (right). For plants grown under low temperature conditions, once bud differentiation was complete, the temperature was returned to the normal growth temperature. When flowers were at stage 7, the proportion of deformed rose flowers was recorded (*n* = 50). **Figure S3.** Silencing of *RhAG* gene caused conversion of gynoecia to sepal-like organs. Top panel, The *RhAG*-silenced and TRV control flowers without petals. The sepal-like gynoecia and normal gynoecia are indicated. Middle and Bottom panels, the sepal-like gynoecia from *RhAG*-silenced flowers. Scale bar, 10 mm in Top panel; 10 mm (left) and 5 mm (right) in Middle panel; 10 mm in Bottom panel. **Figure S4. **Schematic view of the *RhAG* gene structure. Lines represent introns, black boxes represent exons. The first base pair of start codon was defined as 0.The numbers indicate the 3’ ends of the DNA fragment. Bp, base pair. **Figure S5.** Percentage of CG, CHG, and CHH methylation in the *RhAG* promoter. Percentage of CG (A), CHG (B), and CHH (C) methylation was determined by bisulphite sequencing analysis of the *RhAG* promoter, using genomic DNA extracted from flower buds grown at the indicated temperature day/night temperatures. Seventeen clones were analyzed for each treatment. **Table S1.** Oligonucleotide primer sequences. (PDF 645 kb)

## Abbreviations

*AP*: *APETALA*
*PI*: *PISTILLATA*
*AG*: *AGAMOUS*
*SEP*: *SEPALLATA*
VIGS: Virus-induced gene silencing
quantitative RT-PCR: quantitative reverse transcriptase polymerase chain reaction
cDNAs: complementary DNAs
